# The Hypopiezotolerant Bacterium, *Serratia liquefaciens*, Failed to Grow in Mars Analog Soils under Simulated Martian Conditions at 7 hPa

**DOI:** 10.3390/life10060077

**Published:** 2020-05-26

**Authors:** Andrew C. Schuerger, Rebecca L. Mickol, Petra Schwendner

**Affiliations:** 1 Space Life Sciences Laboratory, Department of Plant Pathology, University of Florida, 505 Odyssey Way, Exploration Park, Merritt Island, FL 32953, USA; petra.schwendner@ufl.edu; 2 American Society for Engineering Education, 1818 N St NW #600, Washington, DC 20036, USA; rebecca.mickol@gmail.com

**Keywords:** forward contamination, extant Mars life, astrobiology, human missions to Mars

## Abstract

The search for life on Mars is predicated on the idea that Earth and Mars life (if present) should be both carbon- and water-based with similar forms of evolution. However, the astrobiology community can currently only investigate plausible Martian microbial ecosystems by using Terran life-forms as proxies. In order to examine how life might persist on Mars, we used a hypopiezotolerant bacterium (def., able to grow at 7–10 hPa)—*Serratia liquefaciens*—in growth assays with four Mars analog soils conducted under a subset of simulated Martian conditions including 7 hPa, 0 °C, and a CO_2_-enriched anoxic atmosphere (called *low-PTA* conditions). The four Mars analog soils included an *Aeolian* dust analog, the Mars *JSC-1* analog, a *Phoenix* lander-site simulant, and a high-*Salts* analog. *Serratia liquefaciens* cells were able to grow at 30 °C in a liquid minimal basal medium (MBM) supplemented with 10- or 20-mM sucrose, Spizizen salts, and micronutrients. When the four analog soils were doped with both MBM and cells of *S. liquefaciens*, and subsequently incubated at 30 °C for 72 h, cell densities increased between 2-logs (*Phoenix* analog) and 4-logs (*Aeolian* and *JSC-1* analogs); the *Salts* analog led to complete inactivation of *S. liquefaciens* within 24 h. In contrast, when the experiment was repeated, but incubated under *low-PTA* conditions, *S. liquefaciens* cells were either killed immediately by the *Salts* analog, or decreased by >5 logs over 28 d by the *Aeolian*, *JSC-1*, and *Phoenix* analogs. The failure of *S. liquefaciens* to grow in the analog soils under *low-PTA* conditions was attributed to the synergistic interactions among six factors (i.e., low pressure, low temperature, anoxic atmosphere (i.e., the *low-PTA* conditions), low-pH in the *Salts* soil, dissolved salts in all analogs, and oligotrophic conditions) that increased the biocidal or inhibitory conditions within the analog soils. Results suggest that even if a hypopiezotolerant Terran microbe is displaced from a spacecraft surface on Mars, and lands in a hydrated and nutrient-rich niche, growth in the Martian regolith is not automatically assured.

## 1. Introduction

The search for life on Mars is predicated on the assumption that Martian life is likely to follow the model of Terran life that includes carbon-based organics, liquid water as a solvent, and is constrained by similar forms of evolution. However, until the scientific community has a confirmed extant Mars microbe or community to study, we are forced to use Terran microbial life as proxies for how life might persist on Mars. In general, it is also assumed that the best proxies for exploring how microbial life might persist and grow on Mars is through the study of microbial communities in extreme environments like the Antarctic dry valleys, the Atacama Desert, alpine sites, oligotrophic niches, and saline and acidic geochemistries like in Rio Tinto, Spain [1,2,3,4,5,6,7]. In contrast, others have proposed that microorganisms recovered from Mars spacecraft should be used as model organisms [8,9,10,11] because they might plausibly be dispersed onto the Martian terrain during lander or rover missions, and thus, might act as inoculum for contaminating sites of scientific interest. 

Recently, a series of studies were published relevant to Mars habitability that demonstrate metabolic activity and growth for a diversity of bacteria and algae under conditions that are ≤100 hPa (see review by Schwendner and Schuerger) [12], and as low as 7 hPa (i.e., 7 mbar) [10,13,14]. Many of the species tested under low pressures ≤100 hPa were bacteria isolated from extremophilic ecosystems in the arctic and regional deserts [15,16,17,18]. Of all of the hypopiezotolerant bacteria (def., as those microbes capable of metabolism and growth at low pressures <10 hPa [12]) discussed in the literature above, the bacteria in the genera *Bacillus*, *Carnobacterium*, and *Serratia* have received the most attention. 

*Serratia liquefaciens* was selected for the current study due to a proven record as a hypopiezotolerant bacterium capable of growth at 7 hPa [10], metabolic profiling at 7 hPa with 95 sole-source organics [19], transcriptomic responses at 7 hPa [13], and tolerance to desiccation and moderate salt levels [20]. Furthermore, *Serratia* spp. have been recovered from spacecraft hardware and clean rooms [21,22,23] and may be present on future robotic or human spacecraft to Mars.

Although the literature cited above suggests a diversity of Earth microorganisms can grow under simulated Mars conditions of 7 hPa, 0 °C, and CO_2_-enriched anoxic atmospheres (henceforth called *low-PTA* conditions), almost all of the literature to date used liquid- or agar-based media for metabolism and growth assays under *low-PTA* conditions. In the few attempts to grow bacteria in Mars analog soils under realistic surface pressures at 7 hPa, the biggest problem encountered was the desiccation of the soils and subsequent loss of microbial survival [1,20,24]. Thus, some attempts have been made to study microbial survival and growth in Mars analog soils at low pressures (≤50 hPa) in which liquid media are added [25,26,27]. However, only a few studies have confirmed bacterial growth at the pressures (7–10 hPa) normally found on the surface of Mars [10,17,19].

The primary goal of the current study was to determine if *S. liquefaciens* is capable of growth (i.e., cell proliferation) in Mars analog soils under *low-PTA* conditions. The research outlined below is based on the following three assumptions: (1) Microorganisms that might be displaced from spacecraft surfaces on Mars will remain viable until they are transported to potential habitable niches, (2) spores or cells are then protected from solar UV irradiation by being quickly emplaced within the regolith, and (3) the potential habitable niches are hydrated and possess nutrients that will support microbial activity. Thus, the primary hypothesis was that the hypopiezotolerant bacterium—*S. liquefaciens*—would grow under *low-PTA* conditions when mixed into hydrated Mars analog soils supplemented with essential nutrients. 

## 2. Methods

### 2.1. Microbiological Procedures 

Cells of the bacterium, *Serratia liquefaciens* ATCC 27592, were maintained on trypticase soy agar (TSA) plates at 30 °C for 18–24 h prior to preparing cell suspensions for the experiments described below. Cell suspensions were mixed in sterile deionized water (SDIW; 18 MΩ) and calibrated to equal ~2 × 10^6^ viable cells/mL with a Genesys 30 Visible Spectrometer (Thermo-Scientific Corp., Madison, WI, USA) set at 600 nm yielding optical densities (OD) of ~0.007 (range 0.005 to 0.010). 

Microbial populations were determined using a previously described most probable number (MPN) assay [9,28] to estimate cell densities on a per-milliliter or a per-sample basis. For cell enumerations, 1 mL of the cell suspensions (in SDIW) were serially diluted and 20 µL per dilution pipetted into separate wells in a 96-well microtiter plate filled with 180 µL of trypticase soy broth (TSB) per well. Each dilution was dispensed into 16 wells (two columns) of the 96-well plates. Plates were incubated at 30 °C for 24–48 h and visually read for the number of positive wells per dilution. 

For Mars analog soils, 5 g of analog soils (with cells) were placed in separate 50-cc polystyrene conical tubes containing 15 mL of SDIW. The soil/SDIW mixtures were agitated for 2 min on a vortex mixer set at maximum, soil particles were allowed to settle for 10 s, then cell suspensions were serially diluted and processed by the MPN procedure.

### 2.2. Mars Analog Soils

Five Mars analog soils were used in the experiments to cover a wide range of geochemical compositions, hydrogen ion concentrations (pH), and electrical conductivities (EC). The Mars analog soils were described previously [1,29,30,31] and were labeled as *Aeolian* (airborne dust component), *Basalt* (base soil; from Duluth, MN, USA), Mars *JSC-1* soil (an Hawaiian palagonite), *Phoenix* (based on the regolith at the Phoenix lander site), and *Salts* (a high-salts analog soil based on the Paso Robles soil, Husband Hill, Gusev Crater, Mars). All analog soils were pre-sterilized at 130 °C for 72 h prior to use. 

**Figure 1:**
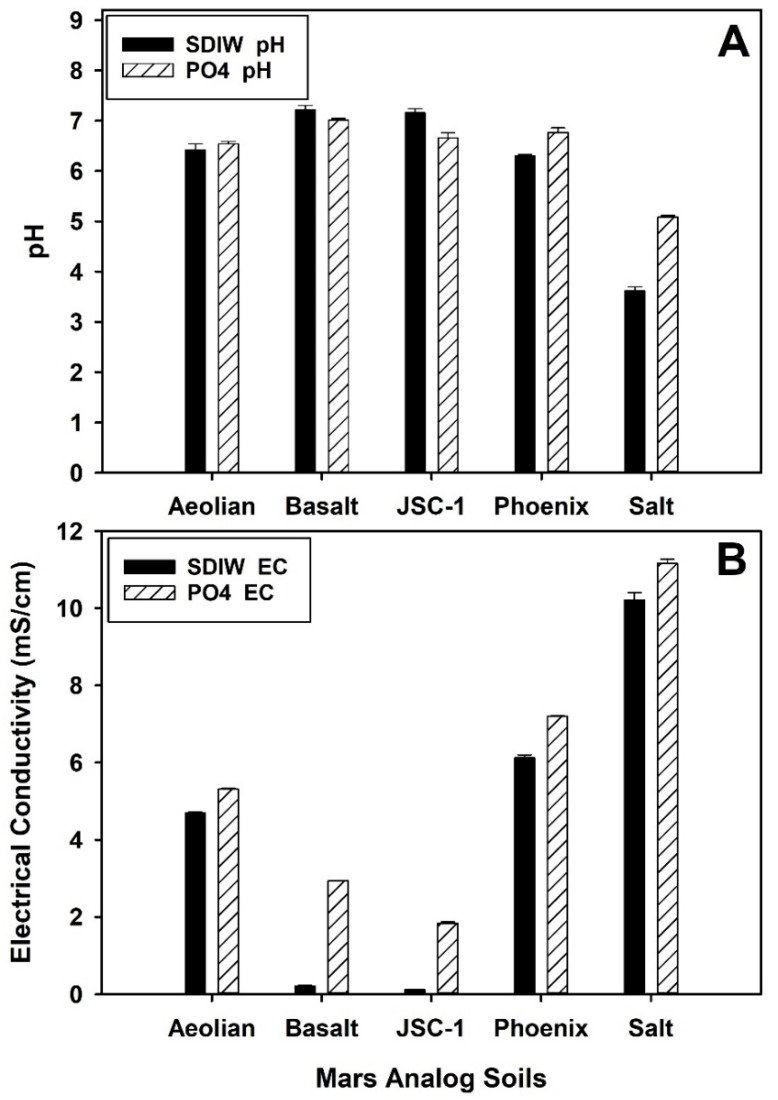
Hydrogen ion concentration (pH) (**A**) and electrical conductivity (EC) (**B**) for all Mars analog soils extracted with either sterile deionized water (SDIW) or 10 mM phosphate (PO_4_) buffer. All soils except the *Salts* soil exhibited pH values between 6.30 and 7.22. In contrast, the *Salts* analog exhibited the lowest pH and highest EC levels of all soils tested. The use of the 10 mM PO_4_ buffer slightly increased the pH for the *Salts* soil but had little effect on raising the pH levels of the other soils. Bars are standard errors of the means; *p* ≤ 0.01; n = 3 per treatment.

The pH and EC of all soils (Figure 1) were determined by mixing 50 g of each soil into separate 100-mL aliquots of SDIW in 250-mL Erlenmeyer flasks, and agitating the soil/water mixtures vigorously at 250 rpm for 2 h on a rotary shaker. The aqueous phases of all soil/water mixtures were filtered first through no. 4 Whatman filter paper on Buchner funnels, and then post-filtered through separate 0.45-µm sterile filters (polyethersulfone membrane, Whatman Puradisc 25 AS, Fisher Scientific, Pittsburgh, PA, USA) to achieve particle-free and sterile soil solutions. All soil solutions were then measured for pH and EC using vendor directions (Oakton PD300 Series, Oakton Instruments, Vernon Hills, IL, USA; and Orion Star A325, Thermo Fisher Scientific, Beverly, MA, USA, respectively). 

### 2.3. Sonication to Enhance Recovery of Cells from Mars Analog Soils

Initially it was hypothesized that sonication might enhance the recovery of *S. liquefaciens* cells from doped soils by helping to dislodge cells weakly attached to soil particles without killing the cells. To test this hypothesis, aliquots of *JSC-1* soil were doped with cells at a rate of ~2 × 10^6^ cells/50-cc conical tube containing 5 g of soil; inocula were prepared in SDIW. Into each 5-g aliquot of soil, 4 mL of SDIW were added, and each tube was inoculated with 1 mL of viable cells. The conical tubes were separated into two cohorts of samples to be sonicated between 0 and 20 min at 37 or 80 kHz using an Elmasonic P sonicator (Elma GmBH & Co., KG, Singen, Germany). Replicate tubes per sonication frequency were placed into the water bath of the sonicator and the kHz set. Samples were removed from the sonication bath at 0, 0.5, 1, 1.5, 2, 3, 4, 5, 10, 15, and 20 min (total n = 24 per frequency). Crushed ice was periodically added to the sonication bath to keep the temperature of the fluid ≤30 °C. Each frequency of sonication was repeated 3 times, and the soils processed by the MPN protocol. The 5-g soil aliquots of *JSC-1* soils were diluted with 15 mL of SDIW, agitated by vigorous vortexing for 2 min, soil particles allowed to settle for 10 s, cell suspensions serially diluted, and then processed as described above. 

### 2.4. Extraction of Bacterial Cells from Doped Soils 

**Figure 2:**
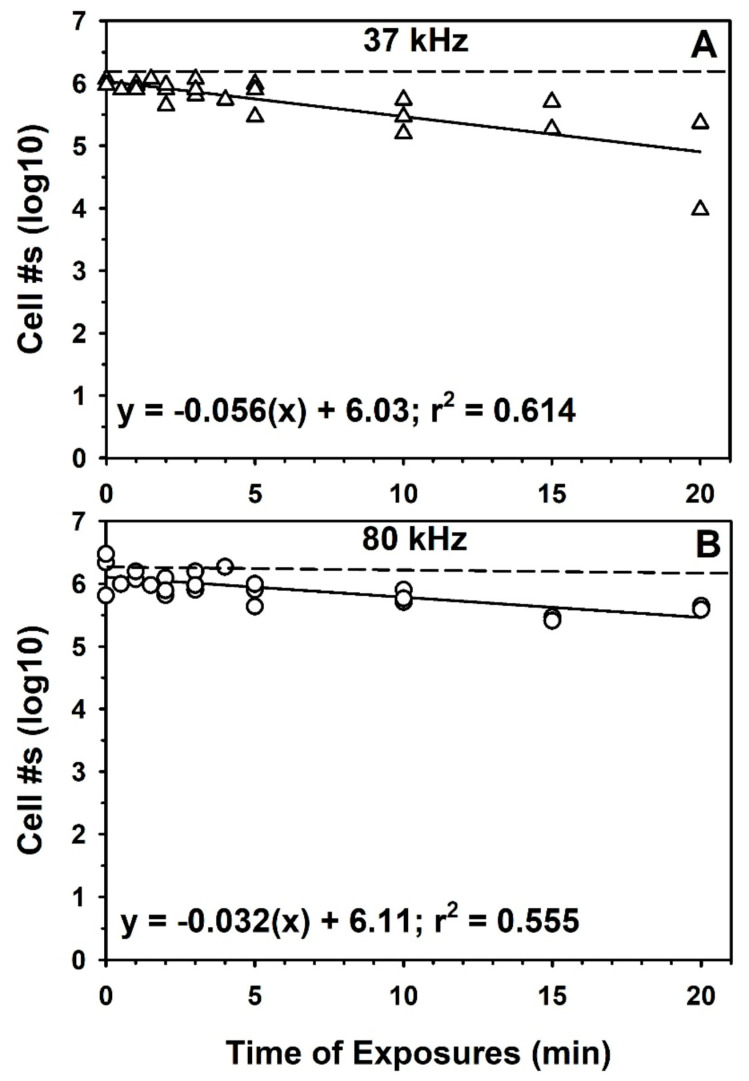
Effects of sonication on the recovery of viable cells of *Serratia liquefaciens* from the *JSC-1* analog soil. Sonication at two different frequencies failed to increase the recovery rates of cells from the *JSC-1* soil. Biocidal effects were observed for both frequencies and resulted in approx. 1-log decreases in viable cells recovered from *JSC-1* samples after 20 min. Data were log-transformed prior to running PROC REG on both datasets. Both linear models deviated from horizontal lines and were significant (*p* ≤ 0.05; total n = 24 per linear model).

As described in more detail in the results section, the sonication of the soils did not increase the recovery of *S. liquefaciens* cells from the doped *JSC-1* analog soil, and thus, sonication was not used in downstream soil experiments. In fact, it appeared that sonication decreased recovery, especially after 20 min, by inactivating cells over time (Figure 2). However, we still required an efficient soil recovery process of viable cells of *S. liquefaciens* from the Mars analog soils if we were to develop a useful soil-growth protocol. 

Stock suspensions of cells were created in SDIW as described above and added to 5-g aliquots of all five Mars analog soils in separate 50-cc conical tubes to yield ~2 × 10^6^ cells/tube. The doped analog soils were mixed with pre-sterilized stainless-steel spatulas (i.e., at 130 °C for 72 h), and immediately assayed with the MPN protocol. The extraction fluids for separate cohorts of conical tubes were either SDIW or 10 mM phosphate buffer (henceforth, PO_4_ buffer; pH 7.0; composed of equimolar concentrations of NaH_2_PO_4_ · 2H_2_O and Na_2_HPO4 · H_2_O). The two extraction fluids were tested to determine if the PO_4_ buffer would moderate the extreme pH levels of some analog soils and increase the recovery of viable cells. The necessary dilutions and arithmetic adjustments were conducted to estimate the numbers of viable cells per tube. The goal was to achieve between 75% and 90% recovery of *S. liquefaciens* cells per tube. 

### 2.5. Growth of S. liquefaciens in a Minimal Liquid Growth Medium 

The next step in developing a Mars analog soil bacterial-growth protocol was to determine the best minimal liquid growth medium required for moderate growth of *S. liquefaciens* at 30 °C. Such a minimal medium could then be used to dope analog soils assuring that growth of *S. liquefaciens* in the soils would not be limited by nutrients. Previously, Schwendner and Schuerger [19] identified a number of sole-source organic molecules that could support the growth of *S. liquefaciens* under simulated Martian conditions of 7 hPa, 0 °C, and a CO_2_-enriched anoxic atmosphere. Of the organics identified, sucrose was reported to be one of the best sole-source carbon molecules that supported moderate growth under simulated Mars *low-PTA* conditions. 

The minimal basal medium (MBM) was composed of 470 mL of a 1× Spizizen salts solution [32], 25 mL of a micronutrient solution (see below), 5 mL of an iron-sulfate solution (1.92 g/L of Fe_2_(SO_4_) plus 3.58 g/L of diethylenetriaminepentaacetic acid; DTPA), and 2.5 g of NaCl. To this basal medium, 0.342 g of sucrose was added to yield a concentration of 10 mM sucrose. The solution was filter-sterilized through 0.2 µm filters (polyethersulfone membrane, Fisher Scientific) to prevent the precipitation of the salts encountered when the MBM was autoclaved. The micronutrient stock solution was composed of the following: MnSO_4_ · H_2_O (0.246 g/L), ZnSO_4_ · H_2_O (0.264 g/L), H_3_BO_3_ (0.576 g/L), CuSO_4_ · 5 H_2_O (0.152 g/L), and molybdenum (0.0074 μM (NH_4_)_6_Mo_7_O_24_). 

The MBM was dispensed into 50-cc conical tubes at a rate of 5 mL per tube; no analog soils were included at this stage. Inocula of *S. liquefaciens* cells were created in SDIW, as described above, and added to the 5 mL of MBM to yield ~2 × 10^5^ cells/mL of MBM. Expecting significant growth of *S. liquefaciens* in the MBM over time, a lower than normal starting concentration of cells was used. Cultures were incubated in the dark at 30 °C for 48 h. Each conical tube was treated as a replicate, and then random tubes were assayed at 0, 24, or 48 h using the MPN protocol (n = 6 per treatment). 

### 2.6. Growth of S. liquefaciens in Mars Analog Soils 

The *Aeolian*, *JSC-1*, *Phoenix*, and *Salts* analog soils (but not the *Basalt* soil) were used to determine if *S. liquefaciens* cells might undergo metabolism and cellular replication in soils at 30 °C under an Earth-normal pressure of 1013 hPa. Five-grams of each soil were added to separate 50-cc conical tubes, mixed with 5 mL of MBM, doped with viable cells of *S. liquefaciens*, and incubated in the dark at 30 °C for 72 h. The starting population of *S. liquefaciens* was ~2 × 10^5^ cells per tube. Three replicates of each soil were prepared for each of three runs, and sampled at 0, 24, 48, or 72 h (n = 9 per time-step). After incubation, 15 mL of SDIW was added to each 50-cc conical tube to increase the hydration of the soils and to suspend cells of *S. liquefaciens* in the liquid phase of the soil solutions. The total volume within the tubes equaled 20 cc that contained 5 g of each soil plus 5 mL of MBM and 15 mL of SDIW as the extraction fluid. The soil/water mixtures were vigorously mixed at high speed with a vortex system, allowed to settle for 10 s, serially diluted, and processed by MPN assays, as described above. After the MPN assays, the numbers of cells counted per tube were adjusted to account for all dilution and arithmetic effects in the assays. 

### 2.7. Growth of S. liquefaciens under Simulated Mars Low-PTA Conditions 

Initial experiments to determine if *S. liquefaciens* could grow in Mars analog soils doped with 10 mM sucrose, Spizizen salts, and micronutrients (i.e., the MBM), and incubated for 28 d under *low-PTA* conditions yielded negative results with no obvious growth observed in any of the analog soils (data not shown). It was hypothesized that either the soils became desiccated and growth rates were halted due to low water activities (*a*_w_) in the soils, the inoculum at ~2 × 10^5^ cells per tube was too low, or that there were inadequate organics for metabolism and growth in the soils under *low-PTA* conditions. 

Thus, the protocols for the Mars simulations in analog soils under *low-PTA* conditions were adjusted by (1) increasing the concentration of sucrose in the MBM to 20 mM, (2) increasing the amount of cells at T = 0 to ~2 × 10^6^ cells per tube, and (3) adding an extra 5 mL of the MBM to each aliquot of 5 g of analog soils in the 50-cc conical tubes. Thus, the total volume of the soil/MBM at T = 0 was increased to 10 cc, which created a saturated soil matrix with approx. 3–4 mm of standing MBM observed above the soil surface. During the 28-d *low-PTA* experiments, the standing layers of MBM would decrease due to evaporating out of the 50-cc conical tubes at low pressures. As required, SDIW was added to individual tubes to bring the MBM layer back to a depth of 3–4 mm during the experiments. 

Mars simulations were conducted on three replicates in each of the two experimental runs (n = 6 per treatment) for the *Aeolian*, *JSC-1*, *Phoenix*, and *Salts* analog soils. The *Basalt* soil was dropped for this assay due its low geochemical complexity compared to that of the other analog soils [1,30]. Soils were doped with viable cells of *S. liquefaciens* (as described above), placed in wire racks, inserted into 4-L polycarbonate vacuum desiccators, and connected to separate low-pressure controllers and vacuum pumps [10,17,19]. Prior to closing each vacuum desiccator, four anaerobic pouches and one anaerobic indicator tablet (AnaeroPack System, Mitsubishi Gas Chemical, Co., Remel/Fisher Scientific, Pittsburg, PA, USA) were placed around the periphery of the 50-cc conical tubes. The vacuum controllers were sealed and flushed for 2 min with ultra-high purity (UHP) carbon dioxide (CO_2_) gas. The low-pressure chambers were then transferred to microbial incubators set at 0 °C, and slowly equilibrated to 7 hPa by lowering the total pressure in increments down to 100, 50, 25, and 7 hPa in 15-min intervals [17]. The tubes were then incubated under *low-PTA* conditions for 28 d.

Every 7 days, the low-pressure desiccators were opened, three random 50-cc conical tubes per analog soil withdrawn, fresh anaerobic pouches inserted back into the desiccators, and the systems sealed and equilibrated to 7 hPa. At each time-step, the analog soils with *S. liquefaciens* cells were assayed as described above with the MPN protocol.

### 2.8. Statistical Methods 

All data were analyzed with the Statistical Analysis System (SAS) version 9.4 (SAS institute, Inc., Cary, NC, USA). Log(10)-transformations were used to induce homogeneity of treatment variances in all datasets. However, because ANOVA cannot process zeroes when log-transformations are used, an arbitrary low value of 0.0001 was assigned to each cell in the datasets that had no detectable cells in the assays after 28 d (see Supplemental Data). Most transformed data were analyzed with ANOVA followed by protected least-squares mean separation tests (*p* ≤ 0.05). However, PROC REG was used to test for linear models with the sonication data given in Figure 2. All data were plotted as log-transformed values, and, where appropriate, the LSmeans results were given as different small letters in the figures. 

The Data Management Plan consists of posting all raw data for the experiments described here as Supplementary Data to this article in the journal *Life*, and depositing the raw data in the University of Florida Institutional Repository (UFIR) at the website https://ufdc.ufl.edu/ufirg/.

## 3. Results

### 3.1. pH and EC of Mars Analog Soils

The pH and EC values of the soil solutions for all Mars analog soils (Figure 1) indicated that most soils exhibited pH levels between 6.0 to 7.2 (i.e., *Aeolian*, *Basalt*, *JSC-1*, *Phoenix* analogs), except for the *Salts* analog which exhibited pH values 2–3 units lower. Furthermore, the use of a 10 mM PO_4_ buffer did not significantly affect the pH levels of most of the analog soils, except for the *Salts* analog soil in which the pH shifted from approx. 3.6 to 5.1 with the PO_4_ buffer. In contrast, EC measurements ranged between 0.12 mS/cm (*JSC-1*, SDIW extraction fluid) and 11.16 mS/cm (*Salts*, PO_4_ buffer extraction fluid). In all cases, the use of PO_4_ buffer as an extraction fluid for soils raised the EC between 1.2 mS/cm for the *Aeolian*, *Phoenix*, and *Salts* analog soils, and 2.75 mS/cm for the *Basalt* analog. The highest EC values were between 10.21 mS/cm (SDIW extraction fluid) and 11.16 mS/cm (PO_4_ buffer extraction fluid) for the *Salts* analog soil. Results for both pH and EC using SDIW as an extraction fluid were similar to a previous report on microbial survival in salt solutions relevant to the Martian surface [30].

### 3.2. Sonication

Previous work [33,34] demonstrated that mild sonication of soils in diverse extraction fluids increased the recovery of soil bacteria. Thus, we attempted to use sonication at 37 and 80 kHz to enhance the recovery of *S. liquefaciens* cells in inoculated analog soils. Both sonication frequencies failed to enhance the recovery of cells from the *JSC-1* analog soil, but instead caused a reduction of recovered cells over time (Figure 2). Both sonication frequencies exhibited significant negative slopes for linear models (*p* ≤ 0.01). Since the water bath in the sonication unit was kept at ≤30 °C, results suggested that the loss of viabilities between 0 and 20 min were not due to increased temperatures, but rather due to the sonication process itself (i.e., via cavitation and mechanical collisions of soil particles). Consequently, sonication was not used for down-stream processing of doped analog soils.

### 3.3. Soil Extraction of S. liquefaciens Cells from JSC-1

Before experiments were developed to measure the growth of *S. liquefaciens* in Mars analog soils, the efficiency of recovering bacterial cells from doped soils had to be determined and calibrated. The lower the extraction efficiency of the protocol, the greater the increase in cell numbers would have been required before concluding that growth had indeed occurred. A key constraint for such soil/growth experiments is that the ending population of microbial cells must be greater than the starting population. Our goal was to develop an extraction protocol that would be ≥75% of the starting population. 

**Figure 3:**
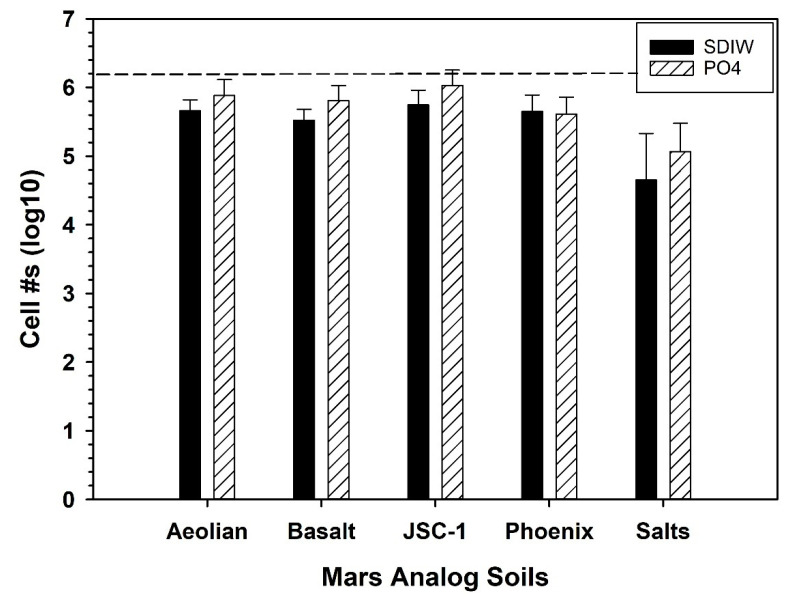
Recovery of viable *Serratia liquefaciens* cells from doped Mars analog soils. Vegetative cells were added to analog soils at a rate of ~2 × 10^6^ cells/50-cc conical tube (see text). Cells were then immediately assayed with a Most Probable Number (MPN) protocol. All simulants yielded between 70% to 85% of the starting populations for the extractions, except the *Salts* analog soil which showed a lower recovery rate between 1–2 logs. Data were log-transformed prior to running ANOVA and protected least-squares mean separation tests. Paired comparisons between SDIW and PO_4_ extraction fluids were not significant (*p* > 0.10). Bars are standard errors of the means; n = 3 per treatment.

Figure 3 shows the extraction efficiencies of recovering *S. liquefaciens* cells from the five analog soils using either SDIW or PO_4_ buffer as the extraction fluid. In all cases, the PO_4_ buffer did not significantly improve the extraction efficiencies of the analog soils compared to SDIW (*p* > 0.10). The extraction efficiencies ranged between 70% and 85% for the analog soils, *Aeolian*, *Basalt*, *JSC-1*, and *Phoenix*. In contrast, a clear negative effect on *S. liquefaciens* cells was observed for the *Salts* analog soil, in which the recovered numbers of surviving cells were reduced by almost 2 logs compared to the other soils. The extractions were conducted within 1 h after the soils were doped with MBM and an average of ~2 × 10^6^ cells per tube. Based on these results, SDIW was used for all downstream extractions of cells from analog soils. Furthermore, the *Basalt* analog soil was dropped at this point in order to focus on analog soils with more complex geochemistries than the base soil (see discussions by Schuerger et al. [1,30]).

### 3.4. Growth in the Minimal Basal Medium (MBM) 

The next step in developing the protocol was to identify a minimal basal medium that would support growth of *S. liquefaciens* without overwhelming the cells with an unrealistic rich and multi-faceted nutrient base. Schwendner and Schuerger [19] identified several sole-source organics that supported active metabolism and cellular replication (i.e., growth) of *S. liquefaciens* under Martian *low-PTA* conditions. Of these compounds, sucrose was selected as the sole-source carbon molecule due to it supporting one of the highest growth rates of *S. liquefaciens* under *low-PTA* conditions compared to 94 other organics. 

**Figure 4:**
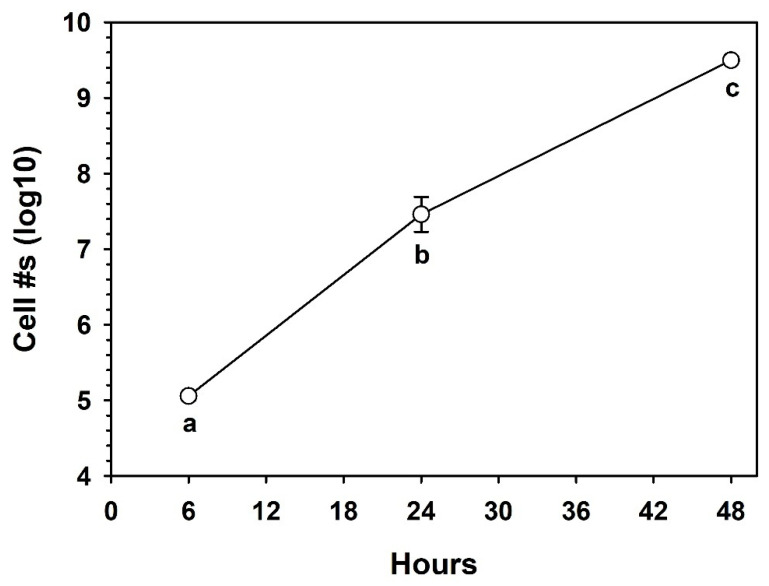
Growth of *Serratia liquefaciens* in minimal basal media (MBM) supplemented with Spizizen salts, 10 mM sucrose, and micronutrients (see text). Growth was immediate and exceeded 4 logs above starting populations (~2 × 10^5^ cells/mL of MBM) after 48 h. Data were log-transformed prior to completing an ANOVA analysis followed by protected least-squares mean separation tests. Treatments followed by the same letters were not significantly different (*p* ≤ 0.01; n = 6 per treatment). Bars are standard errors of the means.

The liquid MBM was inoculated with ~2 × 10^5^ cells/mL. The cultures were incubated in the dark at 30 °C to establish if other micronutrients were required. Initially, trials with only 10 mM sucrose and Spizizen salts failed to promote the growth of *S. liquefaciens* cells in culture (data not shown). It was only when all three ingredients were combined (i.e., 10 mM sucrose, Spizizen salts, and micronutrients) did cell populations increase from ~2 × 10^5^ to > 4 × 10^9^ cells/mL of MBM; a 4-log increase (*p* ≤ 0.01) in cell density (Figure 4). 

### 3.5. Growth of S. liquefaciens in Mars Analog Soils 

Before testing the growth of *S. liquefaciens* under simulated Mars *low-PTA* conditions, we sought to determine if the bacterium could grow in any Mars analog soils doped with MBM. Four analog soils (i.e., *Aeolian*, *JSC-1*, *Phoenix*, *Salts*) were tested at 30 °C and a lab-normal pressure of 1013 hPa. First, populations in the inoculated *Salts* analog soil immediately succumbed to the high salinity and low pH of the soil solutions exhibiting > 5-log reductions in the T = 0 samples assayed at only 20–45 min after adding viable cells to the *Salts* soils (i.e., no viable cells were recovered; data not shown). This result was surprising because in earlier soil extraction assays (Figure 3), cells of *S. liquefaciens* showed only a 3-log decrease after 30–60 min, but some viable cells were recovered. The difference between the two experiments is that for the soil extraction protocol (Figure 3) neither sucrose, Spizizen salts, or micronutrients were added to the soils prior to inoculating them with viable cells. It is possible that in the current experiment, the added nutrients stimulated the cells to attempt active metabolism and growth in the *Salts* soil at higher rates than in the non-nutrient trials (Figure 3), and thus, made the soil solutions more biotoxic to the log-phase cells than expected. 

**Figure 5:**
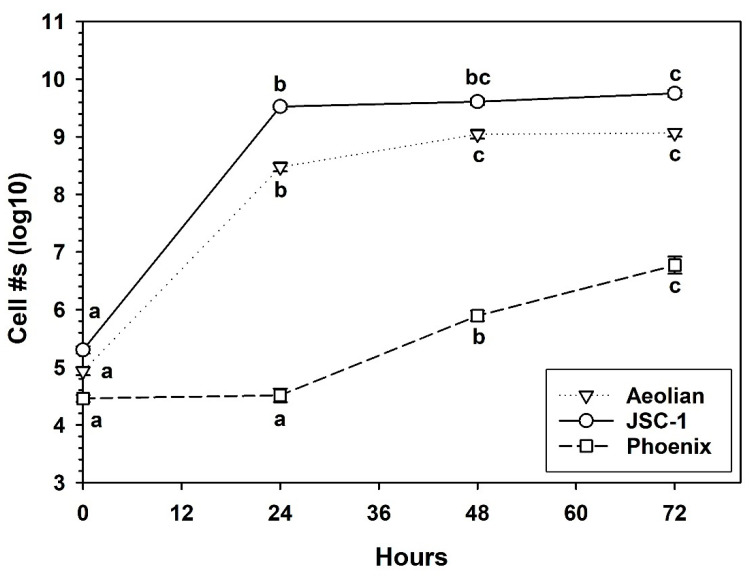
Growth of *Serratia liquefaciens* incubated for 72 h at 30 °C and a lab pressure of 1013 hPa in minimal basal media (MBM) supplemented with Spizizen salts, 10 mM sucrose, and micronutrients (see text). Significant growth of *S. liquefaciens* was observed between T = 0 and 24 h in the *Aeolian* and *JSC-1* analog soils. Cell densities for the *Aeolian* and *JSC-1* analogs increased from ~2 × 10^5^ cells per tube at T = 0 and plateaued when soil cultures reached 8 to 9 logs after 24–72 h. In contrast, growth was delayed for 24 h in the *Phoenix* analog in which the T = 0 and 24-h cultures were approximately similar at 5 × 10^4^ cells/tube, but increased thereafter. Data were log-transformed prior to completing an ANOVA followed by protected least-squares mean separation tests. Treatments for each analog soil (tested separately) followed by the same letters were not significantly different (*p* ≤ 0.01; n = 6 per treatment). Bars are standard errors of the means.

In contrast, the *Aeolian* and *JSC-1* analog soils, exhibited significant increases in cell densities over 24 h that exceeded 3 logs (Figure 5). The growth continued in both analog soils, but appeared to plateau between 24 and 72 h. Interestingly, the cell numbers in the *Phoenix* analog soil exhibited a 24-h delay before increasing cell densities at 48 and 72 h. The delay suggests that the *Phoenix* soil was partially inhibitory to cells of *S. liquefaciens* that required >24 h to overcome. 

### 3.6. Growth of S. liquefaciens under Simulated Mars Low-PTA Conditions 

**Figure 6:**
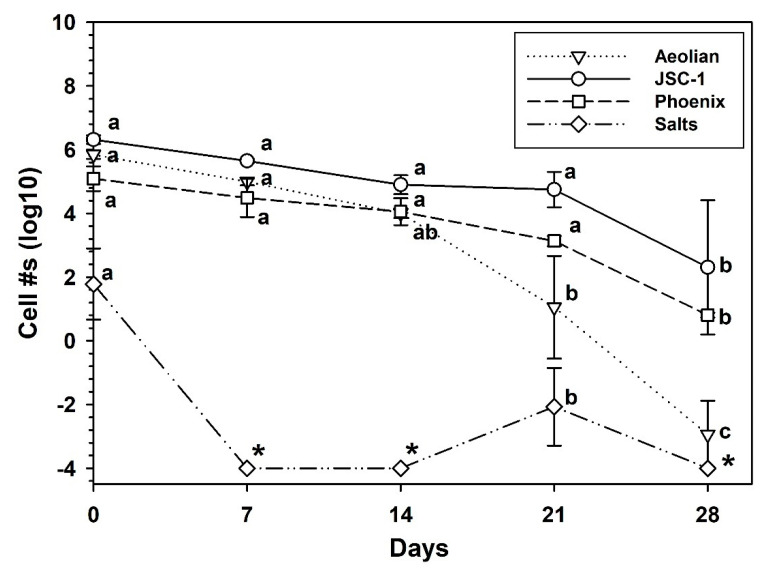
Survival of *Serratia liquefaciens* cells in Mars analog soils incubated for 28 d under *low-PTA* conditions. Analog-soil cultures were supplemented with Spizizen salts, 20 mM sucrose, and micronutrients (see text). The *JSC-1*, *Aeolian*, and *Phoenix* analog soils yielded lower cell numbers over time that approached 2–6 log reductions between the start of the experiment (~2 × 10^6^ cells/tube) and 28 d. The most dramatic decrease in cell population was observed for the *Salts* analog soil in which there was an immediate 4-log reduction for the T = 0 assays (approx. 1 h from mixing to assay) followed by no viable cells recovered in the 7-, 14-, or 28-d cultures (asterisks). Data were log-transformed prior to completing an ANOVA followed by protected least-squares mean separation tests. Treatments for each analog soil (tested separately) followed by the same letters were not significantly different (*p* ≤ 0.01; n = 6 per treatment). Bars are standard errors of the means.

Following the development of the basic protocol that would permit the growth of *S. liquefaciens* in at least three Mars analog soils (Figure 5) under lab conditions, a new series of experiments was initiated to extend the growth studies in the analog soils to *low-PTA* conditions (i.e., 7 hPa, 0 °C, CO_2_ atmosphere). Results for the *Aeolian*, *JSC-1*, *Phoenix*, and *Salts* analog soils indicated that none of the analog soils supported growth of *S. liquefaciens* under *low-PTA* conditions (Figure 6). There were no viable cells recovered from the *Salts* analog soil after 7, 14, or 28 d cultures, suggesting that the *Salts* soil was biotoxic to *S. liquefaciens*, and was consistent with the data in the 30 °C and 1013 hPa experiment. For the 21-d *Salts* replicates, only five total viable cells in two of six replicates were recovered, suggesting that small niches within the *Salts* soil replicates (i.e., 50-cc conical tubes) were not exposed to the extreme conditions encountered by all other cells. For the *JSC-1*, *Aeolian* and *Phoenix* analog soils, cell densities decreased systematically from T = 0 controls (~2 × 10^6^ cells/tube) for soils incubated under *low-PTA* conditions for 28 d. The decreases in all four soils were between 2 and 6 logs (Figure 6). 

## 4. Discussion

The majority of Mars simulations with Terran microorganisms have studied the survival of desiccated cells or spores of bacteria, fungi, and algae at pressures down to 7 hPa (see reviews by Olsson–Francis and Cockell [35]; Schwendner and Schuerger [12]). Attempts to investigate metabolic activity, growth, cellular replication, and evolution at reduced pressures are more limited [10,18,27,36]. One key difficulty with conducting low-pressure simulations close to the surface pressures on Mars (range 2 to 12 hPa) is keeping the growth medium hydrated. The triple point of water on the Martian surface is 0.01 °C at 6.1 hPa, with a very narrow window of pressure (3 and 12 hPa) and temperature (0 and ~10 °C) to maintain pure water in a liquid state [37]. Liquid brines can suppress the freezing points of water, but then microbial activity and growth can be impaired by the presence of specific salt ions and the osmolarity of the brines [38,39]. Thus, Mars simulations with diverse analog soils must consider constraints in thermodynamics, nutrition, biotoxicity, and the stability of liquid water (or brines) in order to accommodate the requirements of microbial metabolism and growth. 

The aim of the current research was to develop a Mars analog soil and microbial growth protocol in which a known hypopiezotolerant bacterium (i.e., *S. liquefaciens*) was given a minimal basal medium (MBM) that supported growth in a series of analog soils with diverse geochemistries, and incubated under simulated Mars *low-PTA* conditions. Our initial hypothesis was that *S. liquefaciens* would grow in at least some of the Mars analog soils over the course of 28 d under *low-PTA* conditions because it had previously been shown that *S. liquefaciens* could grow on TSA after 14 d under *low-PTA* conditions [10]. Furthermore, we observed obvious growth of *S. liquefaciens* in MBM at 30 °C (Figure 4), and in three of the analog soils at 30 °C (Figure 5). Thus, the experiments were designed such that double the incubation-times were allowed for observing positive growth compared to earlier experiments. 

Based on a high extraction efficiency for recovering viable cells from analog soils (75%; Figure 3), we expected to observe obvious growth of *S. liquefaciens* between 14 and 28 d in the most benign soil, the *JSC-1* analog, and to lesser degrees in the other analogs. Surprisingly, all four analog soils exhibited varying levels of biocidal activity resulting in complete inactivation of cells by 7 d (*Salts*), or slow decreases in the recovered cell densities over 28 d (*Aeolian*, *JSC-1*, *Phoenix*) (Figure 6). Although no data were collected here that might reveal if the lower cell numbers at 28 d were due to dormant cells that then failed to germinate during the MPN assays, we believe that the lower cell densities at 28 d were due to death and not dormancy because in other studies [10,14,17], diverse bacteria were reported to remain inactive during *low-PTA* incubations but would reactivate quickly when cultures were returned to normal Earth conditions of 1013 hPa and 30 °C. 

Results did not support the conclusion that growth of the hypopiezotolerant bacterium—*Serratia liquefaciens* ATCC 27592—occurred in any of the four Mars analog soils incubated under *low-PTA* conditions. The conclusion is based on the criterion that soil-dilution assays require, at minimum, a 1-log increase in cell densities over starting populations to indicate active growth (i.e., increased cell numbers over time). This criterion is based on the reported experimental error of approx. ½-log precision in the MPN assays [9,40], and an extraction efficiency of 75% observed here (Figure 3). It is plausible that some cells in all analog soils might have absorbed water/nutrients and divided, but the combined soil-dilution and MPN assays were not precise enough to observe those processes unless ≥1 log increase in cell numbers were observed over time. For example, growth of *S. liquefaciens* was obvious in the *Aeolian*, *JSC-1*, and *Phoenix* analog soils when incubated for 72 h at 30 °C because between 2-log (*Phoenix*) and 4-log (*Aeolian*, *JSC-1*) increases in cell densities were observed at 72 h compared to starting populations of cells. Thus, Mars analog soil growth experiments are tightly constrained by the precision of the assays used. In contrast, if the microorganism being tested produces metabolic or growth by-products that can be measured independently of cell proliferation (e.g., through real-time PCR of 16S rRNA genes, transcriptomics, metabolomics, or gas evolution), it may be plausible to measure metabolic activity and growth without enforcing the ≥1-log increase in cell density rule.

Why would cells of *S. liquefaciens* not grow under *low-PTA* conditions in analog soils when growth was previously shown for both *low-PTA* conditions [10] and in the analogs tested here (Figure 5)? Several papers on the habitability of the Martian surface [6,10,41] discuss between 17 and 22 biocidal or inhibitory factors on Mars that are likely to impact the survival and growth of spacecraft microorganisms. As more and more complex microbial growth experiments are developed, additional biocidal or inhibitory factors are added to the experimental designs. For example, in earlier studies with *S. liquefaciens* [10], *Carnobacterium* spp. [14], and diverse bacteria from arctic soils and permafrost [17], the growth conditions used richer media than used here, and the bacteria were incubated on the upper surfaces of TSA plates free of soil particles. In addition, the analog soils themselves can act as physically constraining materials that can decrease gas and fluid diffusion rates. 

The experiments described here tested Mars analog soils with diverse dissolved salts, pH values, levels of osmolarity not previously tested, and enhanced stress at 0 versus 30 °C. The pH range for *S. liquefaciens* is between 5.55 and 10 [42,43], and thus, it is unlikely that a neutral pH level alone in the *Aeolian*, *JSC-1*, or *Phoenix* soils (Figure 1A) was the primary inhibitory factor in suppressing growth in the soils. It also does not seem plausible that the moderate to low EC values in most of the soils (Figure 1B) would be the primary inhibitory factor for the *Aeolian*, *JSC-1*, or *Phoenix* soils, because *S. liquefaciens* has been shown to tolerate increased salinities between 5% and 10% of diverse salts present on Mars when incubated under lab-normal conditions of pressure (1013 hPa) [20]. However, it is likely that synergistic interactions among the six factors listed above (i.e., low pressure, low temperature, anoxic atmospheres (i.e., the *low-PTA* conditions), low-pH in the *Salts* soil, dissolved salts in all analogs, and oligotrophic conditions) increased the biocidal or inhibitory conditions within the analog soils. Further experiments to factor out the growth-limiting factors in analog soils under *low-PTA* conditions are required.

Of the 14 papers reviewed by Schwendner and Schuerger [12] for growth in low-pressure environments (≤100 hPa), all of the studies met the criterion of ≥1-log increase in cell densities observed over time. In contrast, the study by Pavlov et al. [44] testing the growth of a *Vibrio* sp. under *low-PTA* conditions failed to satisfy the ≥1-log increase in cell numbers over time (i.e., ending populations of cells were 2 logs lower than starting populations of cells), and thus, the conclusion that growth occurred between 0.01 and 1 hPa is equivocal. It is more likely that Pavlov et al. [44] demonstrated only reduced microbial survival and not growth in the test materials exposed to low pressures (0.01 to 1 hPa) and diel temperature fluctuations from −73 °C (nighttime lows) to 27 °C (daytime highs). Again, it is possible that some cells did in fact absorb nutrients and water that led to cell division, but soil assays alone are not precise enough to capture those processes except when the ending populations are statistically higher than the starting populations, and generally by at least ≥1-log. 

Evaluating the results here, and those reviewed elsewhere [12] on microbial growth in low-pressure environments relevant to Mars, the following recommendations are offered as an approach to developing standardized protocols. First, the *low-PTA* conditions used here of 7 hPa, 0 °C, and CO_2_-enriched anoxic atmospheres represent a reasonable global average for the surface of Mars, and should be considered essential test factors in future Mars-relevant growth experiments. If the pressures are much higher than the range found at the surface, the experiments begin to simulate not a surface environment but deeper subsurface conditions. Higher pressures are acceptable for Mars-relevant growth experiments, but the context of where such pressures might exist should be clearly stated.

Second, confirmed hypopiezotolerant microorganisms (e.g., *Carnobacterium* spp., *Exiguobacterium sibiricum*, *S. liquefaciens*, and *Trichococcus pasteurii* [17]) should be used to ensure that at least one positive control for growth be included in all assays of new species within the pressure range (2 to 12 hPa) found on the Martian surface. For example, Smith et al. [45] studied the survival characteristics of *Psychrobacter cryohalolentis* K5—a halophilic and psychrophilic bacterium—to simulated Martian conditions at 7 hPa and exposed to Mars-equatorial UV irradiation. Results clearly indicated survival—but not growth—if the cells were shielded from the simulated solar UV irradiation on Mars. However, in subsequent experiments, Schuerger et al. [10] demonstrated that *P. cryohalolentis* could not grow under low pO_2_ nor low-pressure (7 hPa) conditions. Thus, *P. cryohalolentis* would not be a good candidate microorganism for growth experiments under simulated Martian conditions near 7 hPa. 

Third, the nutrient regime used for simulated Martian growth experiments must be constrained due to the general oligotrophic conditions present in surface fines. Most macro- and micro-nutrients required for microbial growth [46] have been identified in Martian meteorites [47,48] and from in situ measurements [49,50]; and recently, nitrates [51], phosphates [49,52], and organics [53,54] have been discovered in Martian surface fines. However, the organics are likely not ubiquitous, and they might be composed of compounds not easily accessible by Terran microorganisms. Thus, growth assays with hypopiezotolerant microbes, incubated under *low-PTA* environments, should be developed with oligotrophic (i.e., low-carbon) nutritional conditions in mind. 

Fourth, positive growth must be at least twice the precision of the assays used. As described above, we propose that positive growth should only be claimed for soil-dilution assays if the recovered cell densities are ≥1 log higher than the initial populations. Soil dilution assays are not precise enough to measure growth when cell numbers are unchanged or decrease over time; other protocols (e.g., PCR, transcriptomics, metabolomics, and gas evolution) must be employed to avoid false positives for growth when cell numbers do not increase over time. 

## 5. Conclusions

The results presented here have implications for both mitigating the forward contamination of Mars and the search for an extant Martian microbiota. First, forward contamination is a significant concern for both the robotic and human exploration of Mars because inadvertent microbial contamination and proliferation of explored terrains could impact the scientific integrity of the sites. Thus, spacecraft are cleaned and their parts are sterilized during the assembly and launch process to reduce the bioburdens at the launch. The results here are positive news for mitigating forward contamination risks for Mars because even if a hypopiezotolerant Terran microbe is displaced from a spacecraft surface and lands in a hydrated and nutrient-rich niche, growth in the Martian regolith is not automatically assured. To date, approx. 30 bacteria in 10 genera have been identified as hypopiezotolerant microorganisms capable of cell proliferation on diverse media under *low-PTA* conditions [10,14,17]. If any of these hypopiezotolerant bacteria were to find their way onto Mars-spacecraft surfaces, previously we would have hypothesized that they were potential forward contamination risks if cells/spores were displaced into hydrated and nutrient-rich niches on the Martian terrain. However, based on the results here, there might be no increased risk of accidental transport of hypopiezotolerant microorganisms to Mars because other biocidal and inhibitory factors may act synergistically to inhibit cell proliferation in the local terrains around the landers.

Second, the results here suggest that the hurdles that an extant microbial community on Mars (if present) will have to overcome to achieve metabolic activity and cell proliferation may be much more complex and difficult than earlier work with Terran hypopiezotolerant bacteria indicate. It is presumed that if microbial life has persisted on Mars since earlier epochs, such species or communities will have adapted to the conditions found at the surface or shallow subsurface. However, the results here clearly demonstrate that synergism among diverse factors on Mars can make the conditions much more inhibitory, or even biocidal, than any one factor might predict.

Future work will explore the lower limits of growth of other hypopiezotolerant microorganisms under simulated Mars-relevant conditions in the pressure range of 2 to 12 hPa, temperature range of 0 to 5 °C (i.e., to maintain liquid water at low pressures), under simulated Mars atmospheric gas mixtures that represent modern day Mars [55], and oligotrophic conditions (as used here). The protocols developed here are sound, even though no obvious growth was measured for *S. liquefaciens* in four Mars analog soils tested under *low-PTA* conditions. For this reason, new experiments are planned to extend the *low-PTA* incubations up to 60 d, use different hypopiezotolerant species, and test new minimal media to determine whether it is possible for hypopiezotolerant microbes to colonize hydrated analog soils under simulated Martian conditions. 

## Supplementary Materials

The following are available online at https://www.mdpi.com/2075-1729/10/6/77/s1, Raw data for the experiments.
